# Association between *PDCD1* Gene Polymorphisms and Risk of Systemic Lupus Erythematosus in Three Main Ethnic Groups of the Malaysian Population

**DOI:** 10.3390/ijms16059794

**Published:** 2015-04-29

**Authors:** Kek Heng Chua, Lay Hoong Lian, Xiu Jia Sim, Tien Eang Cheah, Tze Pheng Lau

**Affiliations:** 1 Department of Biomedical Science, Faculty of Medicine, University of Malaya, 50603 Kuala Lumpur, Malaysia; E-Mails: lhlian@um.edu.my (L.H.L.); xjsim_leo804@hotmail.com (X.J.S.); tplau@um.edu.my (T.P.L.); 2 Division of Rheumatology, Department of Medicine, Faculty of Medicine, University of Malaya, 50603 Kuala Lumpur, Malaysia; E-Mail: techeah@um.edu.my; 3 Department of Pathology, Faculty of Medicine, University of Malaya, 50603 Kuala Lumpur, Malaysia

**Keywords:** PD-1, SLE susceptibility, Malaysia

## Abstract

The programmed cell death 1 (*PDCD1*) gene encodes for the PD-1 (programmed death 1) molecule, which negatively regulates self-reactive T- and B-cells in the maintenance of peripheral tolerance. A previous report had shown the development of lupus-like phenotypes in PD-1-deficient C57BL/6 mice, was suggestive to the role of *PDCD1* in predisposing to systemic lupus erythematosus (SLE). Hence, we aimed to investigate the association between *PDCD1* and SLE susceptibility in the Malaysian population. A TaqMan-based real-time PCR was employed to screen for PD1.1, PD1.3, PD1.5 and PD1.6 in both SLE and healthy control groups of 200 samples each. The observed frequency for PD1.5C/C genotype was significantly higher in Indian SLE patients and Malay controls (*p* < 0.01). On the other hand, the PD1.5C/T genotype might predispose the Malays to SLE, but confer a protective effect among the Indians (*p* < 0.01). The PD1.1, PD1.3 and PD1.6 were, however, not correlated to genetic predisposition of SLE in our Malaysian population. In conclusion, PD1.5 variant was significantly associated to SLE susceptibility in our Malaysian cohort. Our failure in replicating the association between other investigated *PDCD1* variants and risk of getting SLE might due to ethnic and geographic variations in the distribution of these genetic variants.

## 1. Introduction

Systemic lupus erythematosus (SLE) is an autoimmune disease where the dysregulated T-cells activation, over-production of auto-antibodies and accumulation of immune complexes contribute to its phenotypes (e.g., photosensitivity, skin rashes, prolonged fatigue) and complications (e.g., lupus nephritis, coronary artery disease and osteoporosis) [1]. Worldwide, the SLE incidence is more prevalent among the Asians, Afro-Americans, Afro-Caribbeans and Hispanic Americans, than the Caucasians [2]. There also exists a sex difference in the prevalence of SLE where a ratio of seven women to men was reported [3]. In Malaysia, the prevalence of SLE varies among the three main ethnic groups, *i.e.*, 57/100,000 for Chinese, 33/100,000 for Malays and 14/100,000 for Indians [4]. Over the past decades, the underlying genetic basis in the development of SLE was unraveled by numerous genetic linkage analyses and association studies [5].

In Malaysia, several potential SLE-predisposing candidate genes involved in immune functions, *i.e.*, genes encode for chemokines, interleukins and their receptors, tumour necrosis factors, T-cell surface receptors, components of the classical complement cascade, as well as major histocompatibility complex have been previously studied [6,7,8,9,10,11,12,13,14,15,16,17]. Adding to this expanding list, our present study investigated the relationship between the programmed cell death 1 (*PDCD1*) gene and the development of SLE, which has not yet been reported in our Malaysian population. *PDCD1* is mapped to chromosome 2q37.3 and encodes for PD-1 molecule, which is an immunoinhibitory receptor of the CD28/B7 family. This PD-1 receptor is inducibly expressed on the activated T- and B cells, as well as myeloid cells. It plays a crucial role in the regulation of peripheral tolerance which involves the inactivation or suppression of self-reactive T- and B-cells through the activation-induced cell death or allergy in preventing autoimmunity [18,19,20]. This was proven when the C57BL/6-*PDCD1*^−/−^ mice presented with autoimmune features resembling to SLE, e.g., lupus-like glomerulonephritis with the deposition of IgG3 and C3, arthritis and splenomegaly [21,22].

Genetic association studies were performed in different populations, *i.e.*, European, Caucasian, Mexican, European American, African American and Han Chinese, to examine the correlation between *PDCD1* gene polymorphisms and SLE susceptibility [23,24,25,26,27]. Nevertheless, the causative role of *PDCD1* gene remains inconclusive owing to inter-study heterogeneity in the SNP studied or allele involved [28]. We therefore, aimed to study four identified *PDCD1* SNPs, *i.e.*, PD1.1 (G>A; promoter region), PD1.3 (G>A; intron 4), PD1.5 (C>T; exon 5) and PD1.6 (G>A; 3'-UTR), in our Malaysian SLE cohort.

## 2. Results

All the genotypes of these biallelic polymorphisms were reported in our population, except PD1.3 which AA genotype was absent (Table 1). The observed genotype frequencies among the unaffected controls were conformed to the Hardy-Weinberg Equilibrium (*p* > 0.05). In this study, the allele and genotype frequencies of all *PDCD1* SNPs were not significantly different between the SLE patient and control groups (*p* > 0.05) (Table 1). We also observed the absence of significant association between SLE and PD1.1, PD1.3, PD1.5 and PD1.6 SNPs, irrespective of the model of inheritance used.

**Table 1:** Allele and genotype frequencies of programmed cell death 1 (*PDCD1*) single nucleotide polymorphisms (SNPs) among systemic lupus erythematosus (SLE) patients and healthy controls.

Allele/Genotype	Frequency, *n* (%)		Fisher’s (*p* Value)	OR (95% CI)
	SLE Patient	Control		
**PD1.1**				
A	156 (39.0)	157 (39.2)	1.0000	0.9896 (0.7449–1.3145)
G	244 (61.0)	243 (60.8)
AA	37 (18.5)	36 (18.0)	1.0000	1.0341 (0.623–1.718)
AG	82 (41.0)	85 (42.5)	0.8393	0.9402 (0.632–1.399)
GG	81 (40.5)	79 (39.5)	0.9187	1.0425 (0.699–1.555)
**PD1.3**				
A	2 (0.5)	2 (0.5)	1.0000	1.0000 (0.1402–7.1343)
G	398 (99.5)	398 (99.5)
AA	0 (0)	0 (0)	1.0000	-
AG	2 (1.0)	2 (1.0)	1.0000	1.0000 (0.140–7.170)
GG	198 (99.0)	198 (99.0)	1.0000	1.0000 (0.140–7.170)
**PD1.5**				
C	273 (68.2)	276 (69.0)	0.8789	0.9658 (0.7164–1.3019)
T	127 (31.8)	124 (31.0)
CC	88 (44.0)	90 (45.0)	0.9199	0.9603 (0.647–1.425)
CT	97 (48.5)	96 (48.0)	1.0000	1.0202 (0.689–1.510)
TT	15 (7.5)	14 (7.0)	1.0000	1.0772 (0.506–2.295)
**PD1.6**				
A	228 (57.0)	216 (54.0)	0.4339	1.1292 (0.8543–1.4926)
G	172 (43.0)	184 (46.0)
AA	72 (36.0)	61 (30.5)	0.2885	1.2818 (0.845–1.945)
AG	84 (42.0)	94 (47.0)	0.3652	0.8166 (0.550–1.212)
GG	44 (22.0)	45 (22.5)	1.0000	0.9715 (0.606–1.556)

Further analysis was performed by stratifying our study cohort into three ethnic groups, *i.e.*, Chinese, Malays and Indians. No relationship was observed between PD1.1, PD1.3 and PD1.6 polymorphisms, and SLE susceptibility in either of the ethnic groups (*p* > 0.05) (Table 2). However, statistically significant difference in genotype frequency of PD1.5 between SLE patients and healthy controls was observed in both Malay and Indian groups (*p* < 0.01) (Table 2). Table 3 shows the association analysis of different models of inheritance for PD1.5 and SLE among the Malays and Indians. The association between PD1.5 and SLE was statistically significant with a *p* value of 0.0003 and OR of 3.50 (95% CI = 1.74–7.05) under the best-fitting over-dominant model among the Malays (Table 3). On the other hand, the Indian group showed a significant inverse association between PD1.5 and SLE under the best-fitting over-dominant model (*p* < 0.0001) (Table 3).

**Table 2:** Genotype frequencies of *PDCD1* SNPs among SLE patients and healthy controls of different ethnics.

SNP	Genotype Frequency, *n*								
	Chinese			Malay			Indian		
	SLE	Control	*p* Value	SLE	Control	*p* Value	SLE	Control	*p* Value
**PD1.1**									
**AA**	28	28	ns	8	7	ns	1	0	ns
**AG**	53	53		27	33		2	0	
**GG**	34	34		35	30		12	15	
**PD1.3**									
**AA**	0	0	ns	0	0	ns	0	0	ns
**AG**	0	0		1	1		1	1	
**GG**	115	115		69	69		14	14	
**PD1.5**									
**CC**	56	46	ns	25	44	**0.0022**	7	0	**0.0063**
**CT**	49	60		42	21	**0.0006**	6	15	**0.0007**
**TT**	10	9		3	5	ns	2	0	ns
**PD1.6**									
**AA**	52	47	ns	18	14	ns	2	0	ns
**AG**	49	55		33	35		2	4	
**GG**	14	13		19	21		11	11	

ns: Non-significant; *p* value in bold: significant.

**Table 3:** Inheritance model analysis of PD1.5 and SLE in Malay and Indian groups.

Inheritance Model	Genotype	Malay			Indian		
		OR (95% CI)	*p* Value	AIC	OR (95% CI)	*p* Value	AIC
Dominant	C/C	1.00	**0.0012**	187.6	1.00	**0.0006**	33.7
	C/T-T/T	3.05 (1.53–6.06)			0.00 (0.00–NA)		
Recessive	C/C-C/T	1.00	0.46	197.5	1.00	0.088	42.7
	T/T	0.58 (0.13–2.54)			NA (0.00–NA)		
Co-dominant	C/C	1.00	**0.0015**	187.1	1.00	**0.0003**	31.1
	C/T	3.52 (1.72–7.22)			0.00 (0.00–NA)		
	T/T	1.06 (0.23–4.80)			1.00 (0.00–NA)		
Over-dominant	C/C-T/T	1.00	**0.0003**	185.1	1.00	**<0.0001**	29.1
	C/T	3.50 (1.74–7.05)			0.00 (0.00–NA)		
Log-additive	-	2.01 (1.12–3.58)	**0.016**	192.3	0.25 (0.05–1.31)	0.071	42.3

*p* Value in bold: significant; AIC: Akaike Information Criterion; NA: not applicable.

## 3. Discussion

Genome wide linkage scanning for SLE susceptibility loci in Icelandic and Swedish families had revealed a favourable linkage to the long arm of chromosome 2 at 2q37 [29]. The new susceptibility locus for SLE, *SLEB2*, was then identified via further mapping in a combined set of Nordic families (Icelandic, Swedish and Norwegian) [30]. In view of the biological role of PD-1 in the regulation of peripheral tolerance, the *PDCD1* gene that is located within this locus might serve as a potential predisposing gene to SLE. The interaction between PD-1 and its ligands, PD-L, was found to negatively co-stimulate and attenuate the auto-reactive T- and B-cells through the inhibition of cytokine production and cell cycle arrest in G_0_/G_1_ phase [31]. The deficiency of *PDCD1* gene expression had been proven *in vivo* to result in inadequate auto-reactive lymphocytes removal and auto-antibodies production [22,31,32]. It was thus evidenced that the PD-1-PD-L pathway is pivotal in maintaining peripheral self-tolerance and preventing autoimmune diseases, e.g., SLE [20].

In our multiethnic sample cohort, the similar distribution of allele and genotype frequencies for *PDCD1* polymorphisms were observed in both SLE and control groups. Our findings corresponded well with three earlier reports on Taiwanese and Caucasian SLE patients, where the efforts to demonstrate the association between *PDCD1* gene and SLE susceptibility were failed [33,34,35]. However, statistically significant association was reported for PD1.5 following further sample stratification of our Malaysian cohort into different ethnic groups. Our association analysis revealed that the PD1.5C/T genotype was significantly associated to SLE susceptibility in Malays with a 3.5-fold increased risk compared to the homozygotes when the over-dominant model was assumed. On the other hand, the heterozygous genotype of this exonic polymorphism was significantly correlated to the Indian healthy controls under the same inheritance model. These differences in SNP association to disease in different ethnics revealed a possible interaction with other genetic or environmental factor in determining the predisposition to SLE [36]. In a study by Lee *et al.* (2009) [28], the relationship between PD1.5C allele and SLE susceptibility was also established among the Europeans.

Of all, the PD1.3 is the most extensively investigated. The PD1.3 G to A substitution was demonstrated to abolish the binding of the runt-related transcription factor 1 (RUNX-1) by altering its binding site. This disruption in *PDCD1* transcription will lead to the hyperactivity of self-reactive lymphocytes and subsequent breakdown of peripheral tolerance. Further evidences had shown the correlation between PD1.3A allele and a decreased expression of PD-1 receptor in SLE patients [23,37]. In addition, the relationship of PD1.3 variant and the surface level of PD-1 receptor on activated CD4+CD25+ T cells also grounded its functional involvement in SLE. These regulatory T cells are crucial in maintaining our body’s self-tolerance by curbing the hazardous activities of self-reactive T cells [37,38].

Despite its strong functional grounds, the association between this intronic polymorphism and SLE was failed to address with sufficient consistency. Prokunina *et al.* and Sanchez *et al.* had demonstrated the association between PD1.3A allele and SLE susceptibility in the European, Mexican, Swedish and Argentine cohorts [23,26,39]. On the contrary, a protective effect of PD1.3A allele on SLE was reported among the Spanish cohort [19]. This variant however, was not associated to SLE in our Malaysian population, as well as previously reported northern Sweden, U.S. and Polish cohorts [40,41,42]. Our failure in replicating this relationship might be due to the relatively low minor allele frequency of PD1.3 variant in our population (0.5%) compared to the Europeans (13%) [43]. Hence, the possibility for the minor allele of PD1.3 to be an important predisposing variant in SLE was excluded owing to its extreme rarity in populations of non-European descent, e.g., 0.008% in Asians [24].

## 4. Experimental Section

### 4.1. Study Subjects

Our study cohort was comprised of three main ethnics of the Malaysian population, *i.e.*, Malays, Chinese and Indians. A total of 200 SLE patients admitted to the University Malaya Medical Centre (UMMC), Kuala Lumpur, Malaysia were volunteered for this case-control association study. Two hundred age- and race-matched healthy individuals were also recruited in the control group. The study protocol was approved by the Medical Ethics Committee (Ref. No. 380.1), and written informed consent was also obtained from the study participants.

### 4.2. Genotyping of PDCD1 Polymorphisms

Venous blood samples were collected and genomic DNA was extracted with a similar DNA extraction approach as reported previously [44,45,46]. Next, the genotyping of *PDCD1* variants was performed via real-time polymerase chain reaction (PCR) using pre-designed TaqMan SNP Genotyping Assays on an Applied Biosystems (Foster City, CA, USA) 7500 Fast Real-Time PCR System (Table 4). The real-time PCR was conducted under universal thermal cycling conditions: denaturation step at 95 °C for 20 s, followed by 40 cycles of denaturation step at 95 °C for 3 s and annealing/extension step at 60 °C for 30 s. The singleplex PCR reaction mixture consists of 5 μL of 2× TaqMan GTXpress master mix, 0.5 μL of 20× pre-designed TaqMan SNP Genotyping Assay, 1 μL of 20 ng/μL DNA template, and deionized water in a total reaction volume of 10 μL.

**Table 4:** Pre-designed TaqMan SNP Genotyping Assays for genotyping of *PDCD1* genetic variants.

SNP	Assay ID for Pre-Designed TaqMan SNP Genotyping Assay
PD1.1	C_57931321_10 ([V]: Allele G; [F]: Allele A)
PD1.3	C_57931290_10 ([V]: Allele G; [F]: Allele A)
PD1.5	C_57931286_20 ([V]: Allele T; [F]: Allele C)
PD1.6	C_172862_10 ([V]: Allele G; [F]: Allele A)

[V]: VIC-Probe; [F]: 6-FAM-Probe.

### 4.3. Statistical Analysis

Allele and genotype frequencies of the studied *PDCD1* SNPs were determined in both SLE and healthy control groups, and subgroup analyses were performed following ethnic stratification. The observed genotype frequencies were tested for concordance to Hardy-Weinberg Equilibrium (HWE) by using a Chi-squared (χ^2^) test. The Fisher’s Exact Test was used to assess the significance of the differences in observed allele and genotype frequencies between the diseased and control group. Odds ratio (OR) with 95% confidence interval (CI) was also determined. The SNP association to SLE was measured on (i) dominant model; (ii) recessive model; (iii) co-dominant model; (iv) over-dominant model; and (v) log-additive model. The Akaike Information Criterion (AIC) was calculated to determine the best-fit model of inheritance.

## 5. Conclusions

Of all the investigated *PDCD1* variants, only PD1.5 was significantly associated to the genetic predisposition to SLE in our Malaysian population. The remarkable ethnic and geographic variations in the distribution of *PDCD1* variants might explain the discordant findings related to its contribution in SLE susceptibility. On the other hand, the lack of consistent association between *PDCD1* gene and SLE in different population background might rule out its direct involvement in disease predisposition, implying instead a possible gene-environment interaction.
